# Development of High Strength Particleboards from Hemp Shives and Corn Starch

**DOI:** 10.3390/ma16145003

**Published:** 2023-07-14

**Authors:** Aurelija Rimkienė, Sigitas Vėjelis, Arūnas Kremensas, Saulius Vaitkus, Agnė Kairytė

**Affiliations:** Building Materials Institute, Faculty of Civil Engineering, Vilnius Gediminas Technical University, Linkmenų Str. 28, LT-08217 Vilnius, Lithuania

**Keywords:** hemp shives, corn starch, chemical treatment, particleboard, bending strength, thermal conductivity, short-term water absorption, flammability

## Abstract

In the current study, high-strength boards for the construction industry were developed from renewable natural resources, fibrous hemp shives, and corn starch. During the research, the influence of the composition of the mixture, the processing of raw materials, and technological parameters on the operational properties of the board were evaluated. The influence of the binding material and the water content on the properties of the molded boards was evaluated. It was established that the rational amount of starch is 15% of the mass of the shives, and the amount of water is 10%. It has been established that with the proper selection of the forming parameters of the board, it is possible to avoid internal disintegration of the structure due to the water vapor pressure, increase the bending strength, and ensure uniform sintering of the board throughout the entire volume. It was found that additional processing of hemp shives can increase bending strength by more than 40%. Furthermore, during the processing of shives by chemical means, soluble substances are washed out, which reduces the density and thermal conductivity of the shives. Selection of a rational level of compression allowed us to increase the bending strength of the boards by 40%. The assessment of all factors made it possible to obtain boards with a bending strength of 40 MPa. The additives used made it possible to reduce the water absorption of the boards up to 16 times and obtain non-flammable boards. The thermal conductivity of the resulting boards varied from 0.07 to 0.095 W/(m·K). The analysis of macrostructure and microstructure allowed us to evaluate the process of the formation of bonds between hemp shives.

## 1. Introduction

Most boards in construction and furniture industry are made from poor wood or wood processing waste as filler and bound with phenol formaldehyde-based binders [1,2]. During the last two decades, many different fillers from renewable resources have been proposed in the scientific literature for the production of boards. Cultivated and un-cultivated plant straw, different wood parts, plant leaves, etc., can be used as fillers [3,4,5,6]. Although most of these fillers are bound with traditional phenol formaldehyde-based binders, other binders have also been proposed. Starch, sugar, sapropel, lignin, protein, etc., can be used as binders [6,7,8,9].

Industrial hemp probably receives the most attention for the production of fillers. This attention to industrial hemp is primarily due to the large amount of dry mass obtained per year from one hectare [10]. Additionally, the greater the amount of mass grown, the greater the amount of CO_2_ that industrial hemp absorbs from the air during growth [11,12,13]. Hemp stalks can be used to obtain fibers and shives. Hemp shives are characterized by a finely porous structure, which allows one to obtain lightweight and low thermal conductivity composites [14,15]. Fibrous hemp has an even greater uniqueness compared to wood: it is characterized by a significantly lower amount of volatile organic compounds [16].

Boards for various purposes have been created from industrial hemp shives for a long time. The resulting properties of the boards vary greatly. This is determined by a whole series of factors: technological parameters, raw materials preparation, raw materials composition, binder selection, etc. [15].

Latvian researchers [17] produced boards from different hemp shive fractions and Kleiberit urea formaldehyde resin by cold pressing. The density of the resulting boards was 270–330 kg/m^3^, the bending strength was 2.4 MPa and the thermal conductivity coefficient was 0.057 W/(mK). To improve board properties such as water resistance, porosity, and visual look, a group of additives were used: water, oil- and alcohol-based colorants, zinc oxide nanopowder, and mineral pigments.

Kremensas et al. [18] created biocomposite boards from hemp shives and corn starch by hot pressing. The compressive stress of the boards obtained at 10% deformation ranged from 2.4–3.0 MPa, a bending strength of 4.4–6.3 MPa, and a tensile strength of 0.23–0.45 MPa. The average density of the boards ranged from 319 to 408 kg/m^3^. The amount of corn starch ranged from 10 to 50% by the weight of hemp shives.

Auriga et al. [19] developed particleboards with a density of 650 kg/m^3^. For the production of the control specimens, only wood particles were used, while in the other specimens, 10 and 25% of the wood particles were replaced with hemp shives. The authors found that replacing part of the wood particles with hemp shives allows lighter and stronger boards.

Alao et al. [20] developed particleboards with a density of 477 to 581 kg/m^3^. For the bonding of hemp shives, different binding materials were used: urea-formaldehyde, formaldehyde-free acrylic resin, and bio-based soy resin. Hemp boards based on soy resin showed the best results in tensile and bending strengths, 0.43 and 13.9 MPa, respectively.

Several authors indicate that different thermal and chemical treatments allow one to wash out various soluble substances and volatile organic compounds from raw materials of plant origin [21,22,23]. This not only ensures the safe operation of the hemp shives products but also improves the adhesion to the binder.

The aim of this work is to develop boards with higher bending strength for use in structural applications. The novelty of this work is that the starch is modified with sodium metasilicate; the ratio of corn starch and metasilicate is evaluated; the effect of pressing, density, starch content, and the chemical treatment of hemp shives on the bending strength is examined, as is the effect of additives on water absorption and flammability; and analysis of the microstructure of the formed boards is performed.

## 2. Materials and Methods

USO-31 fiber hemp shives, corn starch, sodium metasilicate, water, flame retardant, and hydrophobizer were used to prepare the specimens. Fiber hemp was grown in Lithuania, Šakiai district. The 0–5 mm shives fraction was used for the tests. The bulk density of the shives was 110 ± 5 kg/m^3^. Corn starch was produced by Cargill Deutschland GmbH, Krefeld, Germany. Corn starch was mixed with sodium metasilicate. The density of sodium metasilicate was 2.61 g/cm³, the melting point was 72.2 °C, and the amount of particles with a size of 16–30 mm was 99%. The sodium metasilicate supplier was UAB Lerochemas, Klaipėda, Lithuania. The expandable graphite ES 350 F5—manufactured by Graphit Kropfmühl GmbH, Hauzenberg, Germany—was used as a flame retardant. The particle size of the expanding graphite was <71 μm 98.0%, the carbon content was 99.70%, the expansion rate 350–700 cm^3^/g, and the starting temperature was 180–240 °C. Flame retardant was mixed with starch before being introduced into the forming mixture. The Tubiquard 44N non-ionic fluorocarbon resin dispersion was used as a hydrophobizer for the water- and oil-repellent finish of all kinds of textiles. The most common application method is padding mangle, but by adding suitable auxiliaries it can also be foamed and, in this way, applied with good ecological and economic properties. In our case, the hydrophobizer was introduced into the water before it was sprayed into the mixture. The quantity of application depends on the required permanency and the desired effect. The Tubiguard 44N was produced by CHT Germany GmbH, Tübingen, Germany.

The formation mixtures were prepared from the raw materials available, the compositions of which are given in Table 1.

The quantities of all raw materials were calculated from the mass of dry hemp shives. In this work, untreated and chemically treated hemp shives were used. In a previous work [15], it was determined that the rational amount of starch to form boards is 10%. In this work, we chose corn starch amounts of 5, 10, 15, and 20% because the starch was additionally modified with sodium metasilicate and its influence on starch kleisterization is unknown. The amount of water depends on the parameters of the molding form and the thermal treatment mode. From previous works [9,15], we know that an excessive amount of water affects the disintegration of the structure, while an insufficient amount of water leads to incomplete bonding of the binder. In this work, we varied the amount of water from 7.5 to 15.0%.

Untreated and chemically treated hemp shives were used for the preparation of specimens. For the tests, fractions of 0–5 mm fraction were used. The data on the shives processing regime are presented in Table 2.

The binder composition was prepared from corn starch and sodium metasilicate. Raw materials selected in different proportions were mixed with water until a mixture of liquid consistency was obtained. The prepared mixture was applied to two parallel wooden plates, which were compressed with additional weights and hardened for 1 h at a temperature of 160 °C in a drying oven. After curing, the mixture is subjected to a tensile test according to EN 1607 [24] to evaluate the effect of the ratio of raw materials on the adhesive capacity of the mixture. Five specimens were prepared for the tests. The tensile strength of the specimens was calculated according to the Formula (1):

(1) $$\sigma_{\mathrm{mt}}=\frac{F_{m}}{l\cdot b}$$

where F_m_ is the maximum recorded tensile force, in N; l and b are the length and width of the tested specimen, in m^2^.

### Forming the Mat of the Specimens

**Figure 1:**
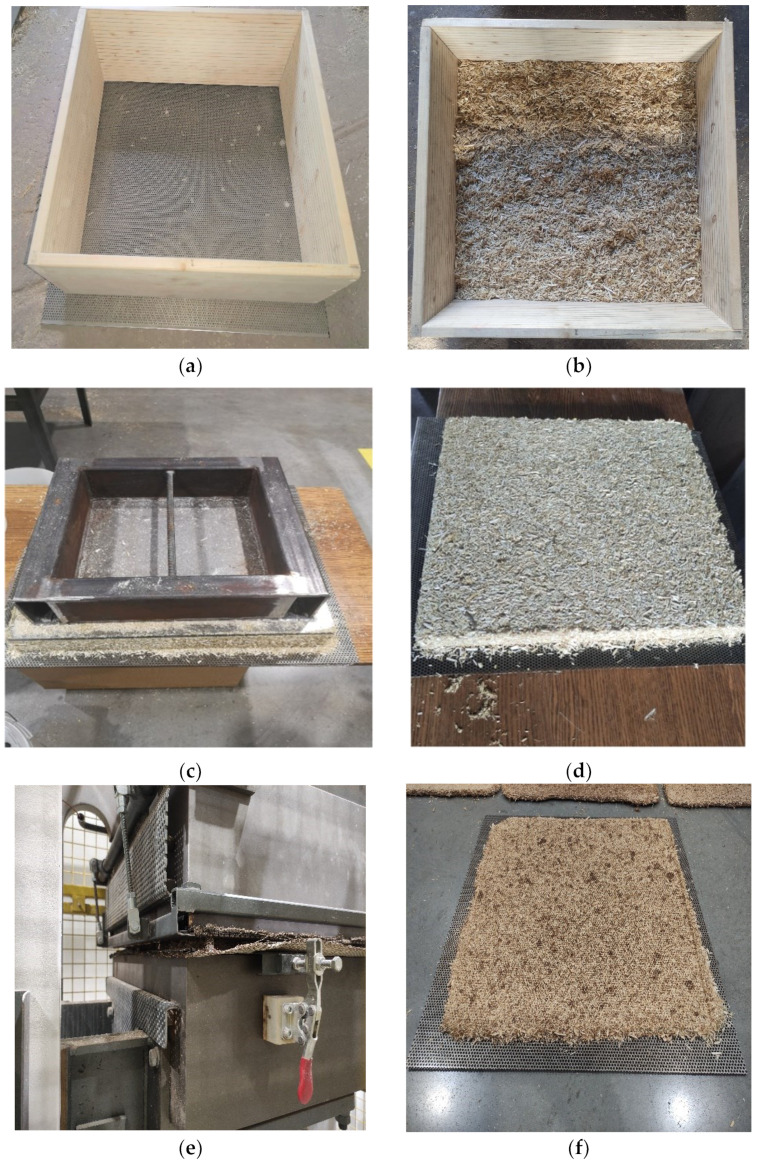
The procedure for preparing the board: (**a**) graduated wooden form on a perforated metal plate; (**b**) the form is filled with the prepared mixture; (**c**) the mat is formed with the help of a pressing plate; (**d**) removed metal pressing plate and obtained board forming mat; (**e**) the mat is pressed with a thermopress; (**f**) the prepared board is removed from the press.

The prepared mixture was poured into a pre-prepared mold (see Figure 1). The mold was placed on a perforated plate, under which there was a stainless steel woven mesh and a protective galvanized plate for the removal of excess moisture during thermal pressing. The poured mixture was leveled, and other layers were formed in the same way. All the poured mixture in the form was pressed with a special cover to form a stable mixture mat.

**Figure 2:**
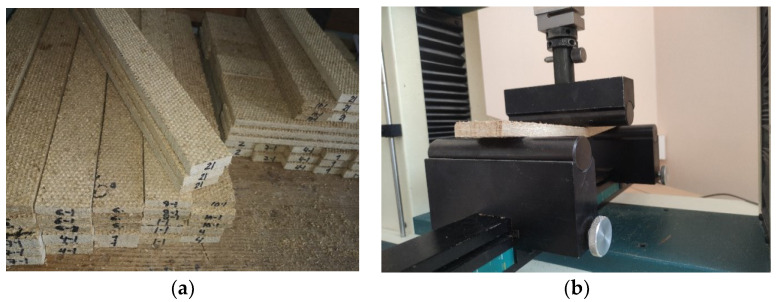
Determination of bending strength: (**a**) preparation of specimens for bending strength tests; (**b**) specimen during testing between press supports.

The main indicator that was taken into account when creating the board was the bending strength. We determined the bending strength according to the methodologies specified in the EN 12089 [25] standard. The test device used for the test consists of two parallel cylindrical roller supports, the lengths of which were greater than the width of the specimen, its diameter is 15 ± 0.5 mm, and a cylindrical loading head with a diameter of 30 ± 0.5 mm was placed parallel to the supports and at the same distance from them (Figure 2). The test was carried out using a computerized press Hounsfield H10KS and the computer software QMat Professional Ver.3.83 adapted to it.

The bending strength is determined by applying a load to the center of a specimen placed on two supports. The specimen is placed on the supports so that its longitudinal axis is perpendicular to the longitudinal axes of the supports and its center is under the cylindrical loading head. During the test, the loading head moves at a constant adjusted speed so that the maximum load force is reached within 60 ± 30 s. The bending strength was calculated by determining the ratio of the bending moment at the maximum load force F_m_ and the moment of resistance of the entire cross-section of the specimen, according to Formula (2):

(2) $$\sigma_{b}={3\;\times \;10}^{3}\cdot \frac{F_{m}\cdot l}{2\cdot b\cdot t^{2}}$$

where σ_b_ is the bending strength, MPa; F_m_ is the maximum load force achieved, N; l is the distance between the supports’ centers, mm; b is the specimen width, mm; and t is the specimen thickness, mm.

Five specimens with dimensions of (20 × t + 50) × 50 × t mm were prepared for the bending strength test. Before the bending test, the prepared specimens were conditioned in an environment with a temperature of 23 ± 2 °C and a relative humidity of 50 ± 5%.

Thermal conductivities were determined for untreated and chemically treated hemp shives as well as for specimens prepared from the boards. Thermal conductivity was determined according to the requirements of the EN 12664 [26] standard by measuring the heat flow and the temperature difference between the surfaces of the specimen. A computerized thermal conductivity device Fox-304 LaserComp was used for the tests. The temperature difference between the measuring plates was 20 °C and they had average temperature of 10 °C. Each specimen was pressed by the device’s vertically moving top plate to ensure good contact between the specimen and the measurement plates.

**Figure 3:**
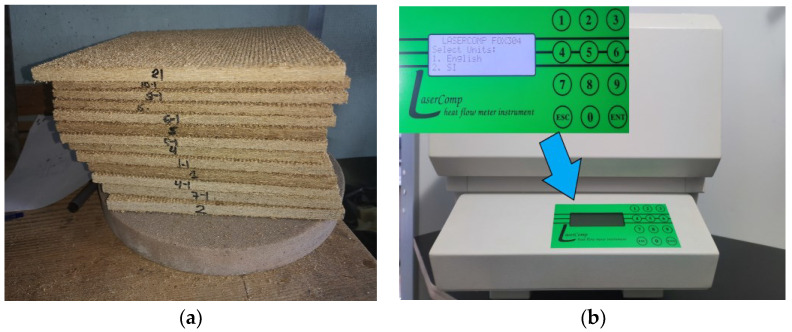
Thermal conductivity studies: (**a**) preparation of specimens; (**b**) determination of the thermal conductivity coefficient of the specimens in the FOX 304 device.

Specimens prepared for thermal conductivity for at least 72 h were kept at a temperature of 23 ± 2 °C and 50 ± 5% relative air humidity. Specimens with dimensions of 300 × 300 × (from 12 ÷ 15) mm were used to test the thermal conductivity coefficient (see Figure 3).

The short-term water absorption of the specimens prepared from the flat prototypes was determined by immersing the specimens in water for a period of 24 h, according to the instructions of method A of the EN 29767 [27] standard. After the specified time, the specimens were removed from the water and excess water that has not been absorbed was allowed to drain. For testing, five specimens with dimensions of 50 × 50 × t mm were prepared from each series of composites. Before the start of the test, the specimens were conditioned for at least 6 h in an environment with a temperature of 23 ± 5 °C. After conditioning, the specimens were weighed, and their lengths and widths were measured. The specimens were placed in an empty bath and a load was placed on them to prevent them from rising when filled with water. Water at a temperature of 23 ± 5 °C was poured into the bath with the specimens until its level reached a level of 10 ± 2 mm above the lower plane of the specimen. The immersed specimens were left in water for a period of 24 ± 0.5 h.

Short-term water absorption after 24 h of soaking in water was calculated according to Formula (3):

(3) $$W_{p}=\frac{m_{24}-m_{0}}{A_{0}}$$

where W_p_ is the short-term water absorption, kg/m^2^; m_24_ is the mass of the specimen after partially immersed in water for 24 h, kg; m_0_ is the initial mass of the dry specimen, kg; and *A*_0_ is the area of the lower plane where the specimen is immersed in water, m^2^.

The combustion tests of the manufactured boards were carried out according to the methodologies specified in the LST EN ISO 11925–2:2010 standard [28]. This test determines the propagation of a small flame up the vertical surface of the specimen when the surface or edge of the specimen is exposed to a small flame for an appropriate period of time. From each series of boards, 5 specimens were prepared with a length of 250 ± 2 mm and a width of 90 ± 2 mm. Before the test, the specimens were conditioned until a constant mass was reached, at least 48 h in an environment with a temperature of 23 ± 2 °C and relative humidity of 50 ± 5%. After the specified conditioning time, the specimen was placed in the holder. The adjusted burner was advanced until the flame reaches the intended point of contact with the specimen. The specimen was exposed to the flame for 30 s. After this time, the burner was retracted. After the set duration of flame operation, the test data were recorded: whether the specimen ignites; whether the top of the flame reaches a height of 150 mm above the point of flame action and after what time; whether there are falling flaming drops or particles that ignite the filter paper; whether the specimen smolders and for how long; and other observations regarding physical changes in the specimen.

**Figure 4:**
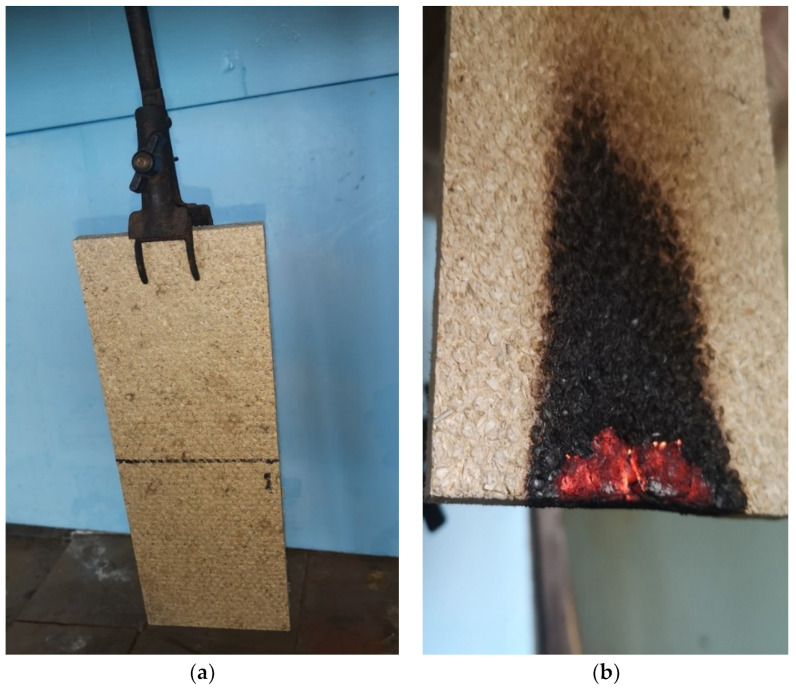
Combustibility tests of the specimens: (**a**) fixation of the specimen in the holder; (**b**) smoldering of the specimen after removing the flame source.

The view of the tests is presented in Figure 4.

## 3. Results and Discussions

**Figure 5:**
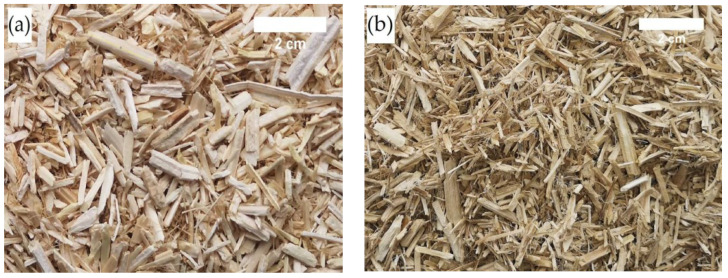
Image of (**a**) untreated and (**b**) chemically treated shives.

An image of untreated and chemically treated shives is presented in Figure 5. After chemical treatment, there was not only the loss of leached materials (see Table 2) but also a change in appearance (see Figure 5b), and many fine particles were formed. These fine particles are hemp fibers that have been attached to the shives prior to processing. During chemical treatment, not only were large amounts of soluble substances washed out but so was a large amount of dust, which negatively affected the board production process: it made the boards heavier and increased the amount of binding material. Hemp shives treated with sodium carbonate and kept in solution for 23 h resulted in a loss of 12.5%.

**Figure 6:**
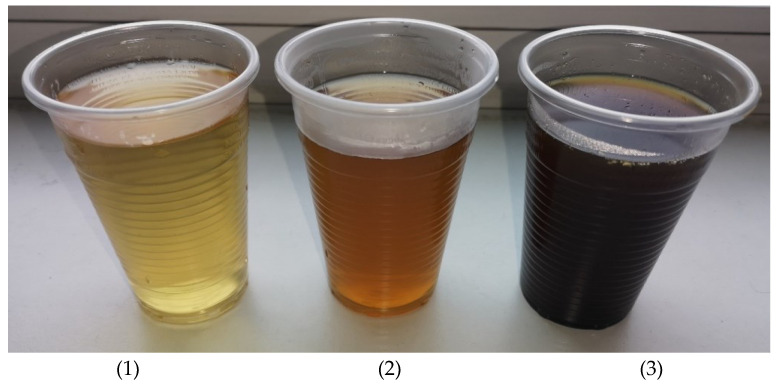
Leaching of the shives boiled in water: 1—after the fifth wash; 2—after the third wash; 3—after the first wash.

According to data presented in the literature, various soluble substances such as sugars, pectin, waxes, paraffin, etc., are washed out with hot water during scallions processing [29,30]. In Figure 6, it can be seen how the leaching of the boiled shives changes in several stages.

**Figure 7:**
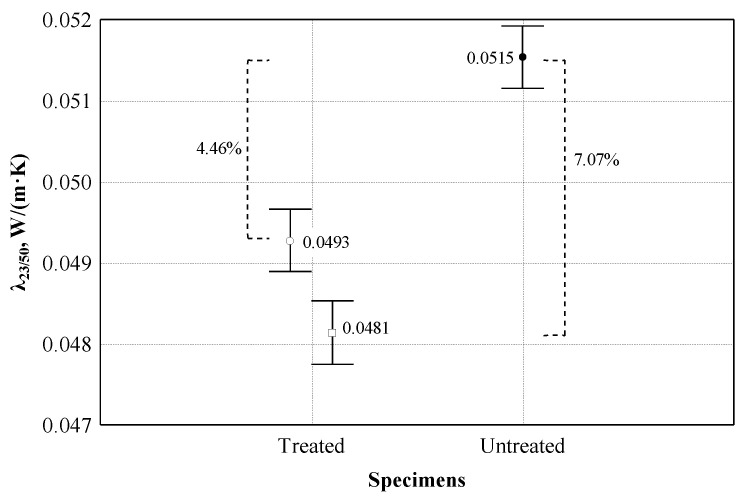
Analysis of the effect of density on the thermal conductivity coefficient: ○—experimental data of treated shives; ●—experimental data of untreated shives; □—corrected data of treated shives.

After chemical treatment of the shives, changes in the density and thermal conductivity of the shives were evaluated. The dispersion analysis conducted [31] of experimental studies showed (see Figure 7) that the averages of the thermal conductivity coefficients of the treated and untreated shives are 0.0493 and 0.515 (W/(m × K)), respectively. The statistic of the F criterion is equal to 91.94, *p* = 0. This indicates a statistically significant difference in the results of the subjects. Consequently, the mean value of the density of the treated shives was (88.0 ± 0.994 kg/m^3^), and the mean value of the untreated shives was (126.8 ± 1.643 kg/m^3^). The coefficient of determination of treated and untreated shives R^2^ = 0.920 was obtained. The bulk density factor of treated and untreated shives explains 92.0% of the variance of the thermal conductivity coefficient in the specimens.

Further studies attempted to equate the bulk densities of treated and untreated shives to assess differences in thermal conductivity. Accordingly, the average value of the bulk density of the shives was (126.2 ± 1.64 kg/m^3^), and the average value of the untreated shives was (126.8 ± 1.64 kg/m^3^). The statistic of the F criterion was equal to 200.73, *p* = 0. This indicates a statistically significant difference in the results of the subjects. The coefficient of determination R^2^ = 0.962 was obtained.

**Figure 8:**
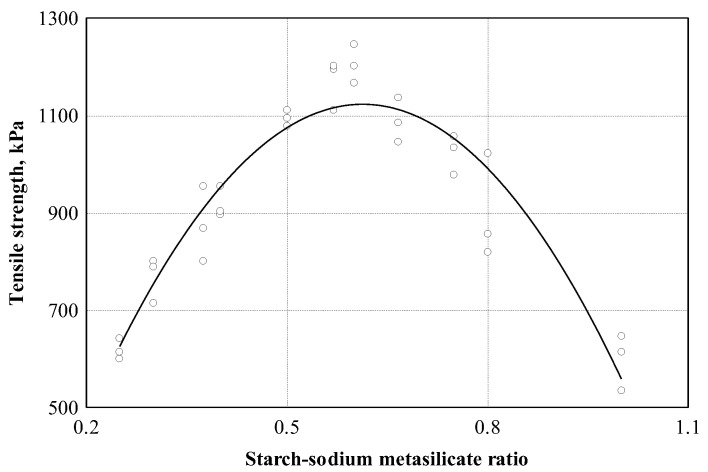
Tensile strength dependence on starch–sodium metasilicate ratio: ○—experimental data; ─── empirical regression dependence.

The main strength Indicator of boards for structural and furniture industry is the bending strength value. The amount of binder is closely related to the bending strength of the boards. In Figure 8, the relationship between the tensile strength of the ratio of starch and sodium metasilicate is shown (see Table 3, Equation (4)). The ratio of starch to sodium metasilicate was evaluated by preparing specimens with different ratios for bending tests.

We can see that the relationship between tensile strength and the ratio of starch to sodium metasilicate is very strong at 0.947. The analysis of the experimental data showed that the variation in the bending strength is 89.8%, dependent on the ratio of starch and sodium metasilicate. For further research, we chose to use a starch–sodium metasilicate ratio equal to 0.6.

**Figure 9:**
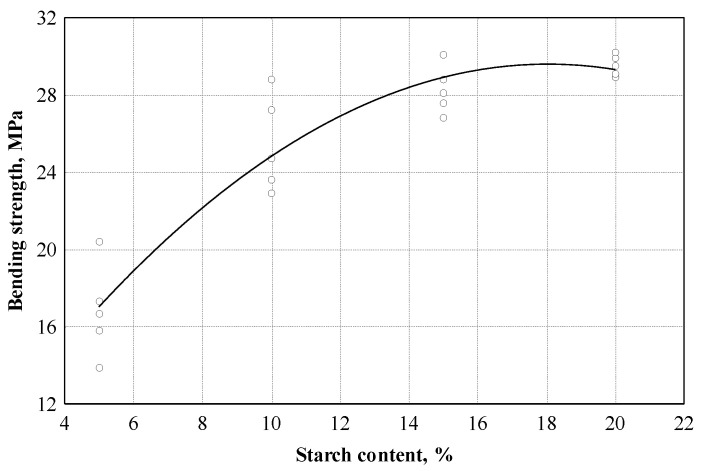
Bending strength dependence on starch content: ○—experimental data; ─── empirical regression dependence.

In the next step, we specify the amount of starch required to obtain the maximum bending strength. The experimental studies carried out showed that the bending strength values of the boards with different starch content can be described according to Equation (5) (see Figure 9 and Table 3).

**Figure 10:**
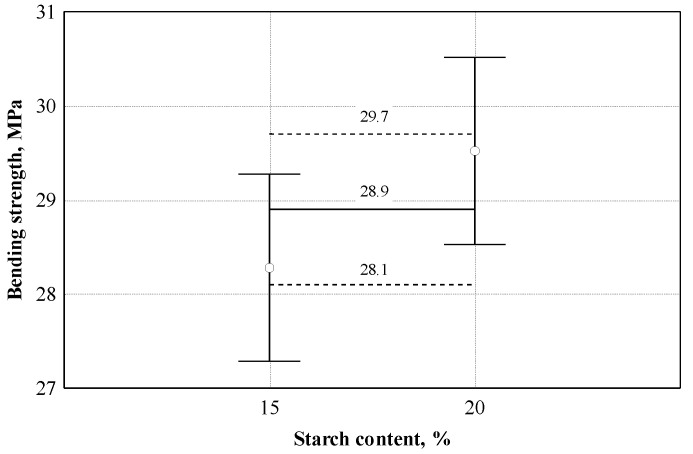
Analysis of the effect of starch content on bending strength: ○—experimental data; ─── average line; - - - - confidence intervals with 0.95 probability.

According to Table 3, the correlation coefficient is equal to 0.944. This shows that the relationship between the bending strength and the starch content is very strong. The coefficient of determination is equal to 0.892; it can be concluded that the amount of starch within the limits of this experiment affects the bending strength by up to 89.2%. To choose the optimal amount of starch for further research, variance analysis was performed to check whether the difference between the obtained results was significant. The dispersion analysis showed that the difference in the average bending strength values was significant; when the amount of starch varied from 5 to 20%, the statistic of F criterion was 47.8, *p* = 0.000. When the amount of starch changed from 10 to 20%, the statistic of F criterion was 8.15, *p* = 0.00581, and the difference in the mean values of bending strength was also significant. Repeated analysis showed (see Figure 10) that when the amount of starch changed from 15 to 20%, the average values of the bending strength do not differ, and the F criterion statistic was 4.12, *p* = 0.0764.

**Figure 11:**
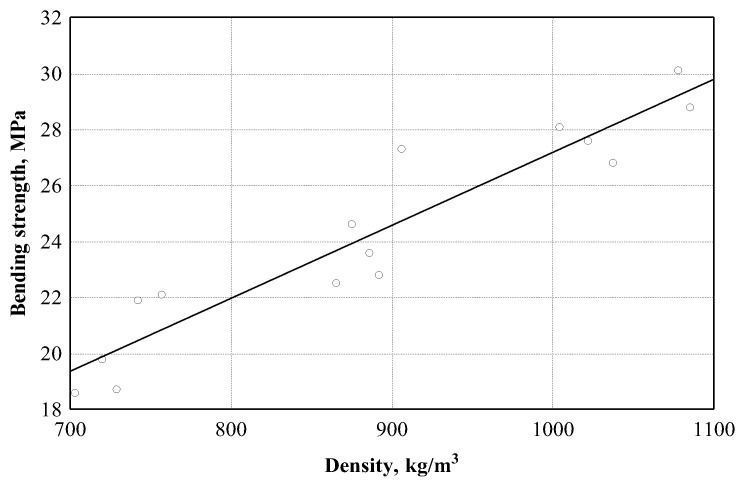
Bending strength dependence on boards density: ○—experimental data; ─── empirical regression dependence.

Figure 11 shows the dependence of the bending strength on the density of the specimens. The density of the boards ranged from ~700 to ~1100 kg/m^3^. On the bases of the experimental data, the relationship between the bending strength and the density of the specimens was determined, the empirical regression equation of which is presented in (Table 3, Equation (6)). We can see that the relationship between the bending strength and the density of the boards is very strong and equal to 0.944. The analysis of the experimental data showed that the variation in the bending strength depends on the density of the boards by 89.1%.

**Figure 12:**
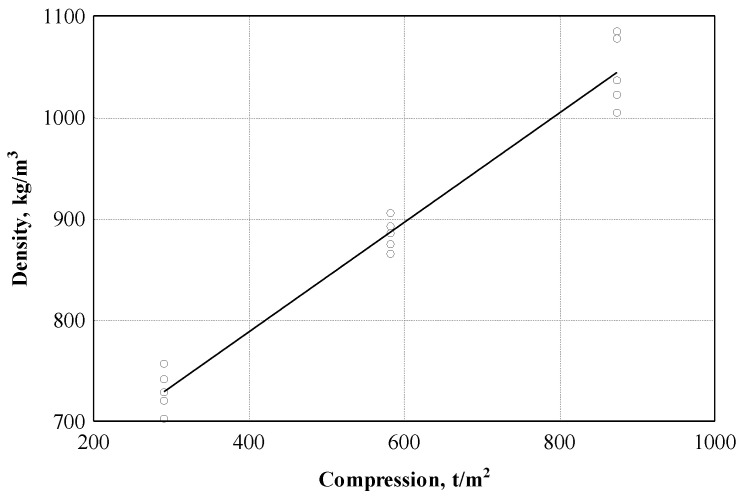
Board density dependence on different compression level: ○—experimental data; ─── empirical regression dependence.

In addition, based on the experimental data, an attempt was made to evaluate the relationship between the density of the material and the compression level. The analysis of the experimental data showed (see Figure 12 and Table 3, Equation (7)) that the variation of the density of hemp shive boards is 97.0%, dependent on the level of compression.

**Figure 13:**
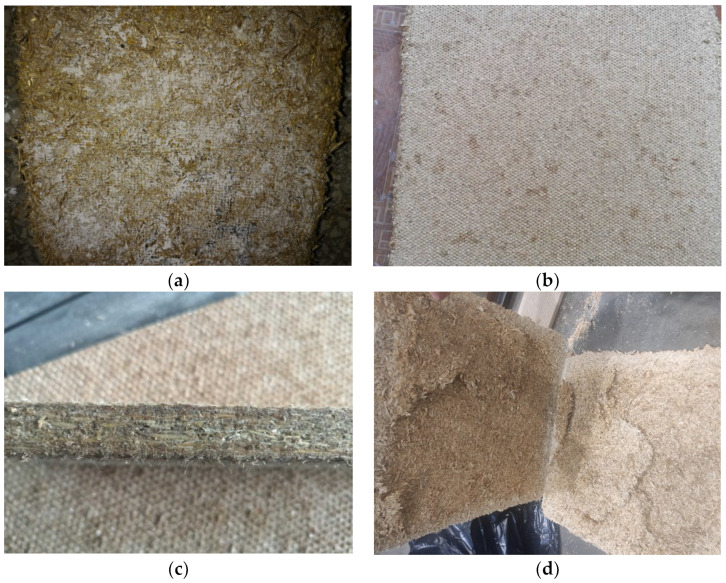
Board view at different water content (in %): (**a**) unbound starch due to lack of water on the surface of the board (7.5); (**b**) free of visible unreacted binder powder on the surface of the boards; (**c**) there are no visible cracks in the middle of the board; (**d**) disintegration of the internal layers of the board due to excessive water content.

In the initial research, we found that the appropriate amount of water is 7.5–15% by the dry mass of shives. The exact amount of water depends on the curing mode and the perforation of the curing plates. For the first tests, the smallest amount of water was used: 7.5%. When changing the curing mode, it was not possible to form the specimens with 7.5% water—the surface of the specimens is formed well, but the specimens are characterized by high brittleness, low bending strength and the powder of unreacted binder is visible on the surface of the boards (Figure 13a). The boards formed with 15% water were free of visible unreacted binder powder but voids formed in the middle of the specimen, resulting in no or weak contact zones in the middle of the board (Figure 13d). With a water content of 10 and 12.5% (Figure 13b,c) on the surface of the boards, there were no spots of unsolidified starch powder or layers in the middle of the board.

**Figure 14:**
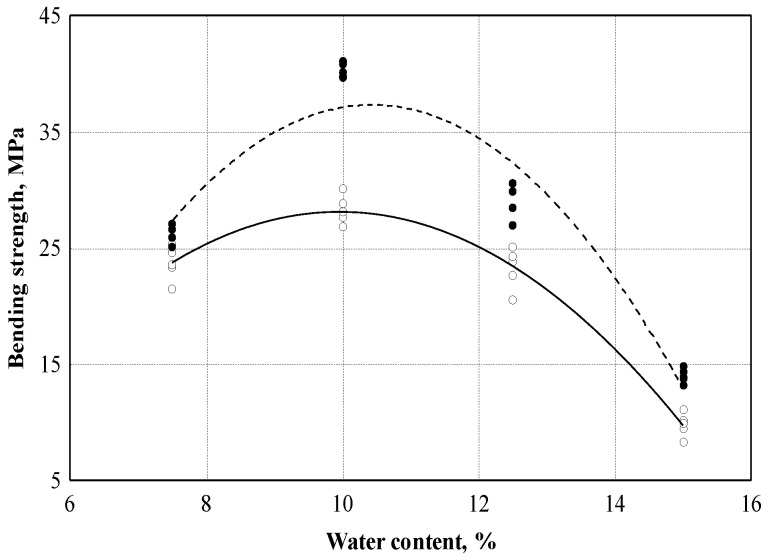
Dependence of the bending strength on water content: ○—experimental data of boards made from untreated hemp shives; ─── empirical regression dependence; ●—experimental data of boards made from treated hemp shives; - - - - empirical regression dependence.

Figure 14 shows the dependence of the bending strength of the boards of treated and untreated shives on different water content, which varied from ~7.5 to ~15.0%. On the basis of the experimental data, a relationship between the bending strength and different water content was determined, the empirical regression equation of which is presented (see Table 3, Equations (8) and (9)). Experimental studies of untreated shives have shown that the variation in bending strength is 96.8% dependent on different water content. Meanwhile, the experimental studies of the treated shives showed that 92.8% of the variation in bending strength depends on the different water content.

With 10% water, the bending strength results were higher, so we chose 10% water for further tests. It is likely that with 12.5% water, the internal decay of the board structure begins.

**Figure 15:**
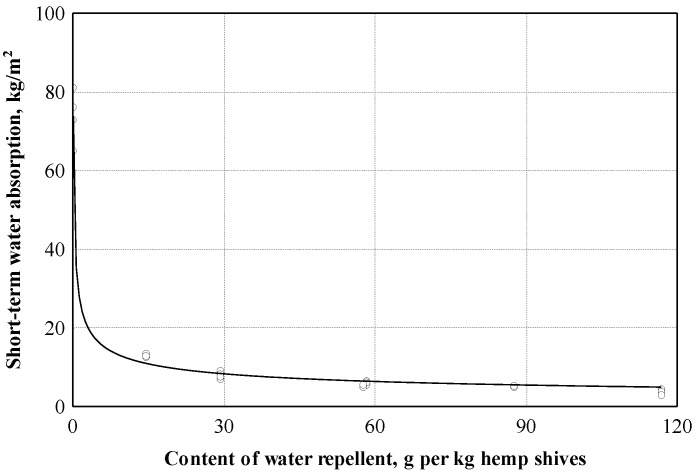
Short-term water absorption dependence on the content of the water repellent: ○—experimental data; ─── empirical regression dependence.

The experimental studies conducted showed that the short-term water absorption values of the hemp shives boards can be described according to Equation (10) (see Figure 15 and Table 3). According to the experimental values obtained, the correlation coefficient is equal to 0.994. This shows that the relationship between short-term water absorption and the amount of hydrophobizing additive is very strong. The coefficient of determination is equal to 0.989, and it can be concluded that the amount of hydrophobizing additive has even 98.9% short-term water absorption. In our research, boards were prepared without hydrophobizer and with different amounts of hydrophobizer, which varied from 15 to 117 g per 1 kg of shives in the mixture. Research results show that even small amounts of hydrophobizer have a significant impact on the water absorption of boards. With 15 g of hydrophobizer, the absorption decreases more than four times. As the amount of hydrophobizer is further increased, there is a slow decrease in the absorbency. In our case, the 117 g amount of hydrophobizer made it possible to reduce the absorption to 3%.

**Figure 16:**
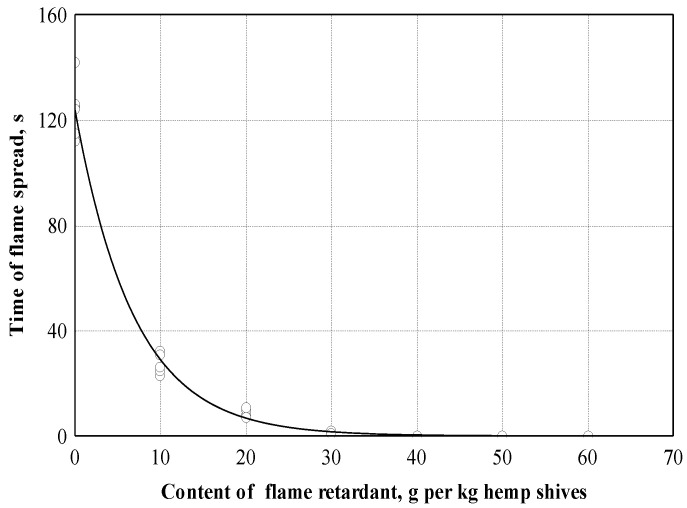
Dependence of time of flame spread on fire retardant content: ○—experimental data; ─── empirical regression dependence.

The experimental flammability studies conducted showed that the fire spread time values of hemp shive boards can be described according to Equation (11) (see Figure 16 and Table 3).

According to the experimental values obtained, the correlation coefficient is equal to 0.995. This shows that the relationship between fire propagation time and the amount of flame-retardant additive is very strong. The coefficient of determination is equal to 0.991, and it can be concluded that the amount of flame-retardant additive even affects the speed of fire spread by 99.1%. The amount of flame retardant was calculated per 1 kg of shives. In our studies, the amount of flame retardant in the mixture varied from 10 to 60 g per 1 kg of shives. A small amount of flame retardant made it possible to significantly reduce the spread of flame: 10 g of flame retardant slowed down the flame spread by about four times, while using a larger amount of flame retardant from 50 to 60 g meant that fire spread did not occur in the boards.

**Figure 17:**
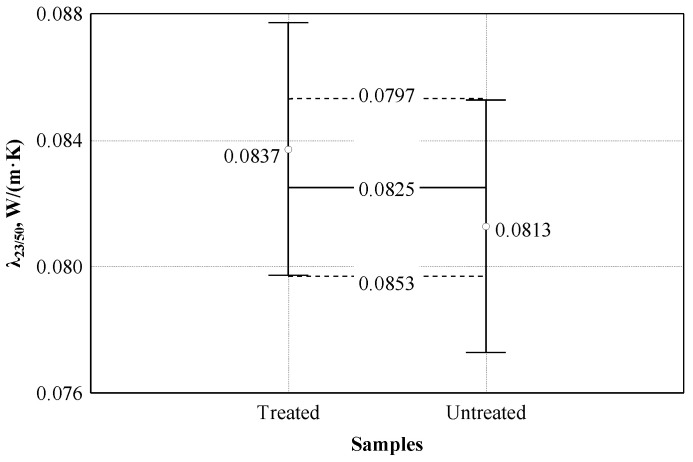
Effect of boards density on thermal conductivity coefficient: ○—experimental data; ─── average line; - - - - confidence intervals with 0.95 probability.

In order to evaluate whether the thermal conductivity coefficient of boards made from differently treated hemp shives differs, a variance analysis was performed. It was checked whether the difference between the averages of the obtained results is significant (see Figure 17). The dispersion analysis showed that the difference between the mean values of the thermal conductivity coefficient is insignificant, and the F criterion statistic was 0.787, *p* = 0.382.

**Figure 18:**
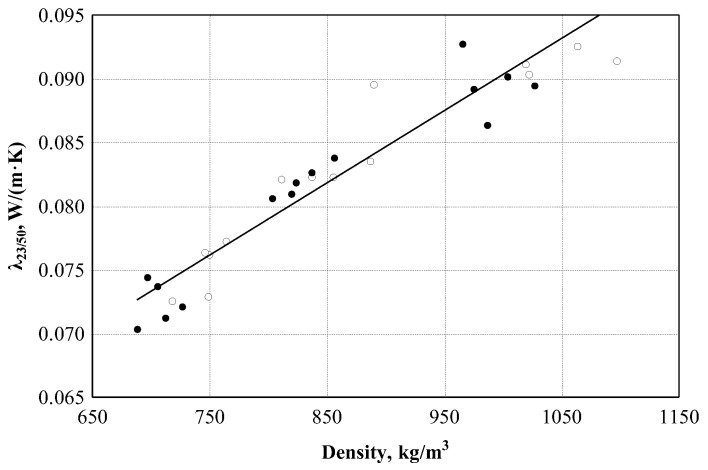
Dependence of the thermal conductivity coefficient on the density of the material: ○—experimental data of boards of treated shives; ●—experimental data of boards of untreated shives ─── empirical regression dependence.

Boards made from treated and untreated hemp shives have no difference in thermal conductivity (see Figure 18). The thermal conductivity coefficients of the boards made from treated and untreated hemp shives were combined and a regression analysis was performed (see Table 3, Equation (12)). Further studies of the experimental data showed that the statistic of the F criterion was 253.9, *p* = 0. Since the *p*-value is less than 0.05, it can be assumed that the regression model is significant. According to Table 3 (see Table 3, Equation (9)), the correlation coefficient is equal to 0.949. This shows that the relationship between the thermal conductivity coefficient and the density of the boards is very strong. It can be concluded that the density of the boards affects the thermal conductivity coefficient by as much as 90.1%.

**Figure 19:**
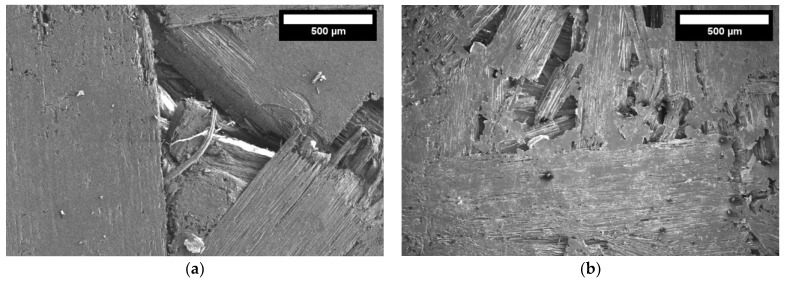

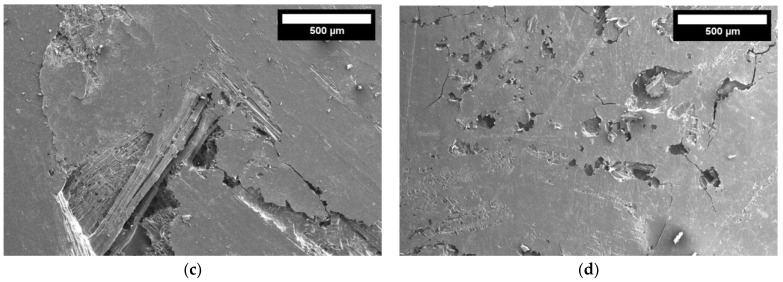
The structure of the board using different amounts of starch (magnification ×100): (**a**) without starch; (**b**) 5%; (**c**) 10%, and (**d**) 15% starch.

In Figure 10 the depicted points can be explained by studies of the boards’ macrostructure. Figure 19a presents the contact zone of fibrous hemp shives without the use of starch. Due to the high pressure, the segments are well pressed against each other, but voids are observed at the intersection of the segments. In the places of compression, partial bonding of the sheets occurs because of the activated binding properties of cellulose at high temperature. The existing voids between the boards have all sorts of negative effects on the strength properties of the boards: there remains a large unbound surface area, a large space for the deformations of the boards during loading, and the existing voids do not meet the requirements for surface smoothness.

After adding 5 or 10% of starch, most of the surface of the boards is bound, there are no longer large voids where deformation would easily occur, and the surface of the board is smoother, i.e., less porous. The starch binder does not completely cover the entire free volume between the slats (Figure 19b,c), but this does not have a major effect on the bending strength of the boards. The existing individual voids prevent the product from easily deforming under loads, so the bending strength changes little.

Further increasing the amount of starch up to 15% completes the filling of individual voids, and the thickness of the starch binder between the layers increases (Figure 19d).

**Figure 20:**
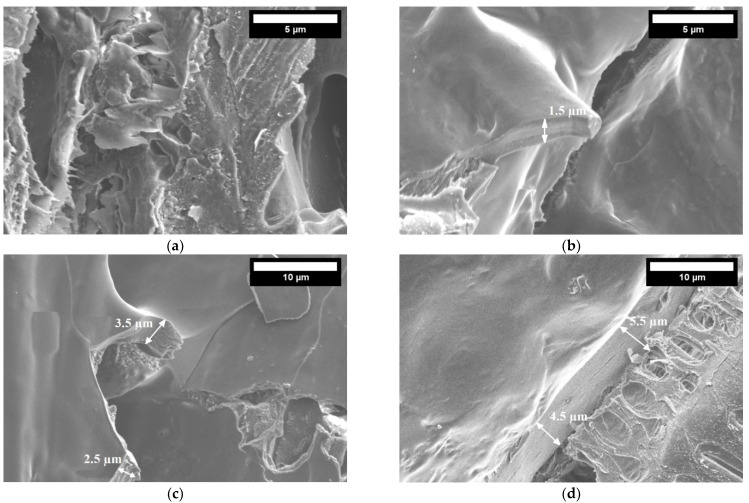
Microstructure of the board using different amounts of starch: (**a**) 5% (magnification ×10,000); (**b**) 10% (magnification ×10,000); (**c**) 15% (magnification ×5000), and (**d**) 20% starch (magnification ×5000).

The microstructure analysis of the board provides information about the contact zones of starch and hemp shives and their quality (Figure 20).

Figure 20a presents a picture of the structure of a board with a 5% binder. In Figure 20a, voids between the starch binder are observed. It can be assumed that, due to the insufficient amount of starch, the binding material contracted and broke during heat treatment, resulting in the formation of elongated opposing binder zones. A very thin thickness of the binding material is observed at the break points of about 0.1–0.3 µm.

By further increasing the starch content to 10%, continuous starch films are formed in individual contact zones. From Figure 20b, it can be seen that the thickness of the film is about 1.5 μm.

Figure 20c presents an image of the structure of a board with a 15% binder. The figure shows that the film thickness is different in individual zones, but it is obviously thicker than with 10% starch: 2.5–3.5 μm.

Meanwhile, Figure 20d shows that with 20% starch, a 4.5–5.5 μm-thickness film is formed in the structure of the board.

The analysis of the results shows that the use of 15% starch provides the required bending strength, while the structure analysis shows that the 15% starch content in the mixture ensures the formation of reliable contact zones.

The works of other authors show that the performance properties of the boards created with starch binding material, especially the bending strength, can differ several times [17,18,19,20,32,33,34]. This is largely due to the density of the panels. In different research works [18,34,35], the density of boards usually ranges from 300 to 800 kg/m^3^. In our work, it was established that when the density is increased by 1.5 times, the strength increases by about 50%. In this case, when the board density is 350 kg/m^3^, the bending strength should be about 10 MPa. In this way, it can be stated that other factors, such as water content, starch content, and starch–sodium metasilicate ratio—analyzed in our work—can lead to an increase in the bending strength of the panels by approximately two times. Similar results are obtained when modified starch is used [32,33,34]. In this case, the bending strength is significantly higher.

## 4. Conclusions

The chemical treatment of hemp shives allowed not only the reduction of the density and thermal conductivity of hemp shives but also the increase of the adhesive interaction between the binder and hemp shives.

Proper selection of the starch–sodium metasilicate ratio allows approximately double the adhesion of the binder. The best starch–sodium metasilicate ratio was found to be 0.6.

It was established that the selection of a rational amount of starch is related not only to the bending strength, but also to the formation of the macrostructure. By choosing a starch content of 15%, the highest bending strength is achieved, and further increasing the starch content does not result in bending strength results. When 15% starch is used, the gaps between the sheets are filled with a binding material, so the surface of the boards is smooth.

The pressing selected during the board production process affects not only the density of the boards but also the strength indicators. If the pressing is increased from 5 t/m^2^ to 15 t/m^2^, the density of the boards increases by about 1.5 times and the bending strength is more than 50%.

The selection of the amount of water allows you to create a board without external and internal defects and additionally increase the strength during bending. A change in the water content of 2.5% increases or decreases the bending strength by approximately 40%. The most suitable amount of water has been determined to be 10%.

It has been found that the use of additives can reduce the water absorption several times and obtain non-flammable boards.

In the future, additional research should be carried out and the coating of boards with additives should be done from the outside of already-finished boards. In this way, the additives would be less negatively affected by the high pressing temperature, and it would be possible to reduce the yield of the additives. In addition, the mixture obtained could be tested by printing with a 3D printer, which should help to obtain an even more continuous structure of the product and better operational properties.

## Figures and Tables

**Table 1:** Compositions of board formation mixtures.

Mixture No.	Hemp Shives, %	Starch/Sodium Metasilicate Ratio	Corn Starch, %	Water, %
1	100	-	5 ÷ 20 (see Section 3)	7.5 ÷ 15 (see Section 3)
2		0.25 ÷ 1.1 (see Section 3)		

**Table 2:** Raw materials and methods used for shives treatment.

Reagent	Duration, Hour	Concentration of the Solution, %	Washing with Water, Times	Losses of Leached Substances, %
Sodium carbonate	24 (boiling for 1 h, stored for 23 h after pouring the boiled solution)	15	6 (after 24 h)	12.50

**Table 3:** ANOVA statistical analysis of results of physical properties.

Equation	Number of Specimens	R	R^2^	Adjusted R^2^	S_r_	F	p
$\sigma_{t}\to S-S.m.r$	$\sigma_{t}=-294.891+4623.208\cdot S-S.m-3769.60\cdot S-S.m^{2}$ (4)						
	33	0.947	0.898	0.891	67.9 (kPa)	132.1	0.000
$\sigma_{b}\to startch\;content$	$\sigma_{b}=5.555+2.664\cdot St.c-0.07380\cdot St.c^{2}$ (5)						
	20	0.944	0.892	0.879	1.89 (MPa)	69.9	0.000
$\sigma_{b}\to density$	$\sigma_{b}=1.0975+0.02608\cdot \rho$ (6)						
	15	0.944	0.891	0.883	1.28 (MPa)	106.4	0.000
ρ→compression	ρ=571.917+0.5403⋅Comp (7)						
	15	0.985	0.970	0.968	24.32 (kg/m^3^)	419.3	0.000
$\sigma_{b}\to water$ ^1^	$\sigma_{b}=-43.634+14.404\cdot W-0.7232\cdot W^{2}$ (8)						
	20	0.984	0.968	0.964	1.37 (MPa)	255.1	0.000
$\sigma_{b}\to water$ ^2^	$\sigma_{b}=-89.554+24.329\cdot W-1.1664\cdot W^{2}$ (9)						
	20	0.963	0.928	0.919	2.73 (MPa)	109.6	0.000
$W_{p}\to Hf$	$W_{p}=30.580\cdot Hf^{-0.3827}\;$(10)						
	24	0.994	0.989	0.987	2.75 (kg/m^2^)	1978.0	0.000
T.fl.sp.→C.f.r	$T.fl.sp.=123.886\cdot \exp\left(-\left(C.f.r\cdot 0.1452\right)\right)\left(11\right)$						
	41	0.995	0.991	0.990	4.18	2147.2	0.000
$\lambda_{23/50}\to \rho$	$\lambda_{23/50}=-0.03363+0.000056703\cdot \rho$ (12)						
	30	0.949	0.901	0.897	0.00241	253.9	0.000

Notes: ^1^ untreated shives; ^2^ chemically treated shives.

## Data Availability

Not applicable.
